# Functional constipation in infancy and early childhood: epidemiology, risk factors, and healthcare consultation

**DOI:** 10.1186/s12887-019-1652-y

**Published:** 2019-08-15

**Authors:** Anne Willemijn Walter, Anne Hovenkamp, Niranga Manjuri Devanarayana, Roshani Solanga, Shaman Rajindrajith, Marc Alexander Benninga

**Affiliations:** 1 Department of Pediatrics, University of Amsterdam, Academic Medical Center, H7-248, Meibergdreef 9, 1105 AZ Amsterdam, The Netherlands; 2 Senior Lecturer in Physiology, Department of Physiology, Faculty of Medicine, University of Kelaniya, Thalagolla Road, Ragama, 11010 Sri Lanka; 3 Medical Officer of Health, Ragama, Sri Lanka; 4 Senior lecturer in Paediatrics, Department of Paediatrics, Faculty of Medicine, University of Kelaniya, Thalagolla Road, Ragama, 11010 Sri Lanka; 5 Department of Paediatric Gastroenterology and Nutrition, Emma Children’s Hospital, Academic Medical Center, Amsterdam, The Netherlands

**Keywords:** Constipation, Infants, Toddlers, Prevalence, Risk factors, Healthcare consultation

## Abstract

**Background:**

Functional constipation (FC) is a pediatric problem that is seen frequently. However, its prevalence in Asia remains undetermined. In this study we attempted to determine the prevalence, risk factors and therapeutic modalities of FC in infants and toddlers in Sri Lanka.

**Methods:**

Children aged 6.5 months to 4 years were selected from 14 well-baby and vaccination clinics in the Gampaha District of Sri Lanka. A questionnaire with questions regarding the socio-demographic characteristics, child’s bowel habits, psycho-social risk factors and treatment modalities were filled by the mothers. FC was diagnosed according to ROME III criteria.

**Results:**

A total of 1113 children were analyzed [(female *n* = 560 (50.3%) with a mean age of 20.7 months, standard deviation [SD] 11.2 months. FC was found in 89 (8.0%). FC was significantly and independently associated with underweight (14.3% vs 7.2%, *p* = 0.008. [OR and 95% CI: 2,3 (CI; 1.3–4.2)] and residence in an urban area (9.6% vs 5.6%, *p* = 0.013). [OR and 95% CI: 0.592 (CI; 0.396–0.95)]. Children subjected to violence showed a significantly higher prevalence of FC (20.0 vs 7.8%, *p* = 0.046). Children being overweight and children living with mothers subjected to violence showed a higher, though not statistically significant, tendency to develop FC. Children with FC visited healthcare clinics more frequently when compared to controls (19.6% vs 6.0%, *p* < 0.0001). However, only 24% of infants and toddlers with FC were treated specifically for the condition by a doctor.

**Conclusions:**

FC occurred in 8% of this cohort of Sri Lankan infants and toddlers. It is significantly associated with underweight and living in an urban area. Only a quarter of them received medical attention for their constipation.

**Trial registration:**

SLCP/ERC/2014/12, December 2014.

**Electronic supplementary material:**

The online version of this article (10.1186/s12887-019-1652-y) contains supplementary material, which is available to authorized users.

## Background

Childhood functional constipation (FC) is a significant health problem [1]. Even in young children the disease negatively affects health related quality of life and leads to considerable healthcare costs [2–4]. It was noted that children under the age of one year had the highest rate of emergency department visits for constipation and related symptoms in the USA and the costs of care had risen by 121% from 2006 to 2011 [5].

Childhood constipation often has its roots in infancy and early childhood. A retrospective chart review of children with constipation revealed that the median age of onset was 2.3 years, with the 25th percentile to 75th percentile range being 0.8 to 4.8 years respectively [6]. An Italian birth cohort study has shown that the prevalence/onset of constipation at 3, 6, and 12 months was 11.6, 13.7 and 10.7%, respectively [7]. A representative community study in the USA noted that 4.7% of infants and 9.4% of toddlers were suffering from functional constipation (FC) [3].

During infancy, the transition from breastfeeding to formula feeding or the introduction of solid foods, is sometimes a trigger for the onset of FC [8]. Furthermore, an association has been suggested between cow’s milk protein allergy and FC. An improvement after a cow’s milk–free diet in young children with FC has been described ranging from 28 to 78% [9]. The latter is supported by evidence of histological changes in the mucosa of the colon indicating inflammation [9].

Poor toilet training in the toddler period is another important risk factor for the development of constipation [10]. When not properly toilet trained, these young children often exhibit stool withholding behavior, which leads to a vicious cycle of stool withholding, pain while passing stools and infrequent bowel motions. Therefore, studying risk factors related to constipation in early childhood may reveal important clues for its etiology and perhaps be helpful in formulating preventive strategies.

Psychological stress is a well-known risk factor to develop FC in older children. Several studies have shown an association between family and school related psychological stressors and constipation in older children [11]. In addition, teenagers who experienced any form of major child maltreatment are also known to develop FC [12]. However, the role of psychological stress and exposure to child maltreatment in developing FC in younger children is not known.

FC is a significant problem in the developing countries as well. A study from Sri Lanka noted that 15.4% of school children had FC [13]. However, there is a dearth of data from developing countries, particularly in Asia, on constipation in infancy and early childhood. Therefore, this study was undertaken with the objectives of a) studying the prevalence of FC in infancy and early childhood, b) identifying risk factors for developing constipation in early life, and c) studying the healthcare consultation patterns of infants and toddlers with FC.

## Methods

### Participants and setting

Subjects were mothers of infants (6.5–12 months), toddlers (13–36 months), and pre-school aged children (37–48 months) who attended well-baby clinics for vaccination and/or growth monitoring in four randomly selected Medical Officer of Health (MOH) areas in the Gampaha District of Sri Lanka. For this cross-sectional study, subjects were selected using the inclusion and exclusion criteria given below.

### Inclusion and exclusion criteria

Children aged between 6.5 to 48 months, living in the Gampaha District and consenting to participate in the study were included. Children were excluded if they had any chronic medical or surgical conditions.

In Sri Lanka, healthcare and immunization services are offered free of charge in the government health clinics. In principle, all babies receive their immunization and basic care during infancy and early childhood in these health centers. A minority (< 1%) receive healthcare in the fee-levying private health care sector. The government clinics are staffed by specially trained staff including doctors, midwives and nurses.

### Sample size

The sample size of the study group was calculated using EpiInfo 6 v60 1996 (EpiInfo 6, version 6.04–1996, Centers for Disease Control and Prevention, Atlanta, Georgia, USA and World Health Organization, Geneva, Switzerland). Following assumptions were used: Estimated prevalence of 10% was expected based on previous literature on childhood constipation. Systematic reviews showed a median prevalence of childhood constipation of 9% [14]. A confidence level of 95%, power of 80% and a precision found to the nearest 2% was used. The sample size for the group estimated using these values was 862 children.

### Questionnaire and data collection

Data were collected using a self-administered questionnaire for mothers, written in the local language (Sinhala). A native language speaking assistant was available to provide any help to the respondents. If any mother had more than one child who fitted the inclusion criteria, she was requested to complete a second questionnaire. Data collection was conducted between February and March 2015.

The questionnaire had 3 sections. Section 1 contained questions on demographics including age, sex, birth order, weight, growth pattern and family size. All children born in Sri Lanka receive a Child Health Development Record (CHDR), which is regularly filled at the well-baby clinic by trained nurses. In Sri Lanka growth monitoring is a mandatory process from birth to 5 years. CHDR was created using the standards of the WHO (WHO muliticenter growth reference study) [15]. Children, whose growth curves were running between +2SD to -2SD, were considered having a normal growth. Children with a growth curve running below -2SD were considered as underweight and children with a growth curve running above +2SD were identified as overweight. Research assistants helped the mothers to select the appropriate answer regarding the growth of their children.

Section 2 contained explicit questions on bowel habits (developed from the Questionnaire on Pediatric Functional Gastrointestinal Symptoms (QPGS) — ROME III version) [16, 17] during the preceding two months. Moreover, this section contained questions about doctor consultation because of stool problems, the tests the doctor had done, and the advised treatment. Treatment could be dietary advice, non-pharmacological interventions, oral laxatives and/or rectal laxatives. For this study, we selected the section on bowel habits and translated it into simple questions that could be easily understood by the mothers.

Section 3 of the questionnaire covered parental education level, employment status of parents and questions on economic and social details. The data collection tool included several stressful life events that could be faced by families. These psycho-social risk factors, such as exposure of mother or child to physical or psychological abuse, economic strains faced by the families and change of residence were questioned in section 2 and 3. A standard questionnaire that has been used in former studies was utilised [12, 18–20].

The final version of the English questionnaire was reviewed by three experts with expertise in field studies on functional gastrointestinal disorders (FGDs) in children (MAB, SR and NMD). Subsequently, it was translated into the local language (Sinhalese) using standard translation/ back translation method by two language experts. The final Sinhala version was reviewed by the aforesaid two local experts (SR and NMD), pretested (AWW and AH) and reviewed for appropriateness. The final version of the questionnaire (in English language) is provided as an additional file (Additional file 1).

### Diagnosis of constipation

Constipation was diagnosed using ROME III criteria for infants and toddlers [10]. Infants and toddlers were considered to have constipation if they fulfilled at least two of the following criteria of FC:

1) Two or fewer defecations per week, 2) At least one episode/week of incontinence after acquisition of toileting skills, 3) History of excessive stool retention, 4) History of painful or hard bowel movements, and, 5) History of large diameter stools which may obstruct the toilet.

### Ethical approval

Ethical approval for the study was granted by the Ethics Review Committee of the Sri Lanka College of Pediatricians.

### Statistical analysis

We used IBM SPSS Statistics for Macintosh, Version 21.0–2012 for the analysis of the data. Characteristics of the sample and prevalence of FC were analyzed using descriptive statistics. Chi-square test was used to detect differences in constipation and controls group in categorical variables, with the confidence level set at 95%. Independent sample t-test was used to analyze differences between means of continuous variables. Tests were two tailed with confidence level set at 95%. *P*-values less than 0.05 were considered statistically significant. The multiple logistic regression analysis was performed using a model which included all variables which were found to have a significant association with functional constipation during univariate analysis. The adjusted odds ratio and independent association between FC and risk factors were determined.

At the initial stage, we analyzed the association between constipation and all categorical variables using the Chi-Square test. Then a multiple logistic regression analysis was performed including all predictive variables that showed association with constipation to identify the independent association between those risk factors and constipation.

## Results

### Sample characteristics

A total of 1300 questionnaires were distributed, of which 1113 (85.6%) properly completed questionnaires were included in the final analysis. The mean age of the children was 20.7 months (SD = 11.2; range: 6.54–47.38 months), 560 (50.3%) were girls (mean age: 20.5 months; SD = 10.9; range: 6.54–46.48 months) and 553 (49.7) were boys (mean age: 20.9 months; SD = 11.5; range: 6.74–47.38 months). Table 1 shows the demographics of the study sample.

**Table 1:** Demographic data of cases and controls

Characteristic	Functional Constipation n (%)	Controls n (%)	*p*-value
Sex			0.477^a^
Girls	48 (8.6)	512 (91.4)	
Boys	41 (7.4)	512 (92.6)	
Age (months, mean (SD))	22.01 (12.6)	20.6 (11.1)	0.254 ^b^
Boys	24.5 (13.6)	20.6 (11.2)	
Girls	19.8 (11.4)	20.5 (10.9)	
Birth order			0.036^a*^
1st	50 (9.6)	470 (90.4)	
2nd	33 (7.8)	389 (92,2)	
3rd	4 (2.9)	135 (97.1)	
4th	1 (3.4)	28 (96.6)	
5th, 6th, 7th	1 (33.3)	2 (66.7)	
Siblings?			0.547^a^
Yes	48 (7.6)	586 (92.4)	
No	41 (8.6)	438 (91.4)	
Place of residence			0.013^a*^
Rural	31 (5.6)	521 (94.4)	
Urban	50 (9.6)	469 (90.4)	
Mothers’ age (years, SD)	30.7 (5.3)	30.6 (4.9)	0.901^b^
Mothers’ education (years, SD)	12.4 (2.7)	12.3 (2.8)	0.773^b^
Fathers’ age (years, SD)	34.0 (6.0)	33.8 (5.2)	0.694^b^
Fathers’ education (years, SD)	12.3 (2.7)	12.2 (2.8)	0.782^b^
Household income (rupee/month)			0.542^a^
< 10.000	8 (11.6)	61 (88.4)	
10.000–19.999	28 (10.2)	246 (89.8)	
20.000–34.999	27 (6.7)	376 (93.3)	
35.000–49.999	17 (7.6)	207 (92.4)	
50.000–99.999	8 (7.3)	101 (92.7)	
> 100.000	3 (7.1)	39 (92.9)	

Legend: ^a^ chi- square test, ^b^ t-test, ^*^ *p* < 0.05

### Prevalence of FC

**Fig. 1:**
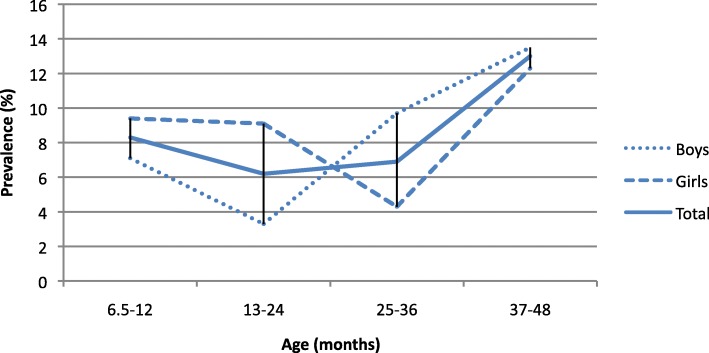
Prevalence of functional constipation according to age groups

A total of 89 infants and toddlers (8%) fulfilled the Rome III criteria for FC. Girls showed a higher prevalence than boys (8.6% vs 7.4%, *p* = 0.477). Children aged between 37 and 49 months showed the highest prevalence of 13% for FC (13.0%). Figure 1 shows the prevalence by the different age groups and sexes. Table 2 depicts children’s bowel habits regarding Rome III criteria.

**Table 2:** Descriptive data of bowel habits of children with FC

Diagnostic criteria of FC	FC n (%)	Controls n (%)	*p*-values
Two or fewer defecations per week	22 (25)	8 (0.7)	< 0.001
At least one episode/week of fecal incontinence after acquisition of toileting skills	24 (27)	26 (2)	< 0.001
History of excessive stool retention	28 (31)	7 (0.7)	< 0.001
History of painful or hard bowel movements	84 (94)	204 (20)	< 0.001
History of large diameter stools which may obstruct the toilet	58 (66)	30 (3)	< 0.001

chi- square test

### Predictors for FC

#### Socio-demographic features

Children living in urban areas of the district showed a significant association with FC compared to children in rural areas (9.6% vs 5.6%, *p* = 0.013). [OR and 95% CI were 0.592 (CI; 0.396–0.95)]. No associations were found with age, being the first-born, having siblings or not, parental age and parental education level.

#### Growth

**Fig. 2:**
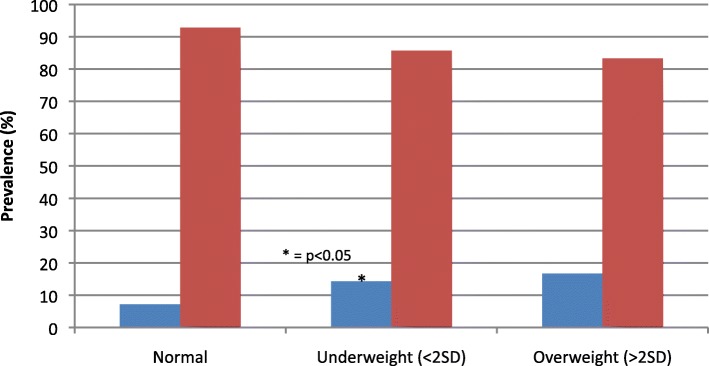
Prevalence of constipation in terms of growth patterns

A total of 69 children (7.2%) with FC had a normal growth curve, and 20 (14.2%) had an abnormal growth curve, *p* = 0.004. [OR and 95% CI were 2.51 (CI; 1.4–4.5), *p* = 0.002]. Of those with an abnormal growth curve, 18 children had underweight (<2SD weight for age). Underweight was significantly associated with FC compared to children with a normal growth curve (14.3% vs 7.2%, *p* = 0.008) [OR and 95% CI were 2.3 (CI; 1.3–4.2)]. Overweight was not correlated with FC (16.7% vs 7.2%, *p* = 0.212). **(**Fig. 2**).**

#### Stressful life events

Table 3 shows the relationships between stressful life events faced by the families and FC. Children subject to physical or verbal violence showed a higher prevalence of FC (20.0% vs 7.8%, *p* = 0.046), but logistic regression analysis did not show an association. Children whose mother suffered physical or verbal violence tend to suffer more frequently from FC than those whose mothers never faced violence (15.1% vs 7.6%, *p* = 0.051). No association was found with shortages in income, family’s loans, quality of relationship between parents and changing the place of residence.

**Table 3:** Stressful events faced by cases and controls

		Functional Constipation n (%)	Controls n (%)	*p*-value
Child exposed to physical or psychological abuse	Yes	4 (20.0)	16 (80,0)	0.046^*^
	No	85 (7.8)	1008 (92.2)	
Mother exposed to physical or psychological abuse	Yes	8 (15,1)	45 (84,9)	0.051
	No	81 (7.6)	979 (92.4)	
Income meets essential needs	Yes	50 (7.9)	580 (92.1)	0.741
	No	38 (8.4)	415 (91.6)	
Family has loans	Yes	37 (8,2)	413 (91.8)	0.770
	No	47 (7.7)	561 (92.3)	
Relationship between parents	Good	86 (8,0)	990 (92,0)	0.751
	Bad	1 (5.9)	16 (94.1)	
Change place of residence	Yes	12 (9.6)	113 (90.4)	0.498
	No	75 (7.8)	881 (92.1)	

Legend: ^*^ *p* < 0.05

### Healthcare consultation

Children suffering from FC visited a healthcare clinic more often because of stool problems than children without FC (36.8% vs 13.1%, *p* < 0.0001). In children with FC, doctors most frequently did either an examination of the abdomen (19.1%) or no test (10.1%). Rectal examination, blood tests and ultrasound examinations were done in 7.9, 4.5 and 2.3% of the children with FC respectively. None of the children were subjected to an X-ray of the abdomen.Forty-seven (52.8%) children with FC, received treatment for their symptoms. Twenty-one of them (24.1%) were treated by a medical doctor and 26 (29.9%) received a number of therapeutic options selected by parents. Table 4 shows an overview of the different types of treatment received by these children. A sizable number of infants (44.9%) and young toddlers were treated with dietary interventions. Another 39.3% received non-pharmacological treatment, and 38.2% were treated with oral or rectal laxatives. Table 5 depicts the number of children, clustered by age, suffering from constipation, who received treatment.

**Table 4:** Treatment of functional constipation

	Number (%) of children suffering Functional Constipation treated with:
Dietary modification	
Total	40 (44.9)
Eating more fibers	17 (19.1)
Eating more fruits	29 (32.6)
Drinking more water	31 (34.8)
Other	5 (5.6)
Non-pharmacological modification	
Total	35 (39.3)
Toilet training	29 (32.6)
Punishing	0 (0)
Rewarding	7 (7.9)
Other	2 (2.2)
Drug treatment	
Total (oral + rectal)	34 (38.2)
Oral laxatives	27 (30.3)
Rectal laxative	17 (19.1)
Herbal remedies	3 (3.4)
Other	2 (2.2)

**Table 5:** Children suffering of constipation clustered by age in months (n (%)

Number (%) of children receiving treatment and the number toilet trained				
Age (months)	6.5–12	13–24	25–36	37–49
Received treatment	18 (38)	10 (21)	6 (13)	13 (28)
Treatment – dietary	17	9	5	9
Treatment – non-pharmacological	16	4	5	10
Treatment – drug	16	4	5	10
Toilet trained (amongst the ones receiving treatment)	7	4	5	7

Not all percentages are shown due to small numbers

## Discussion

This cross-sectional study provides the first epidemiological data about the prevalence of FC amongst infants and toddlers in a developing country. The prevalence rate of FC in infants (8.3%) was higher than that of toddlers (6.6%). Children aged between 37 and 48 months showed the highest prevalence (13%) of FC. Living in an urban area, being underweight and being subjected to physical violence were significantly associated with FC. The majority of children with FC were treated with dietary manipulations and non-pharmacological interventions, while almost 40 % of the children received laxatives.

FC is a common problem in childhood across the world. A similar prevalence, compared to our results, of 8.5% was found in a cross-sectional Korean study. Although, their included age category differed from our study (25–84 months vs 6.5–48 months) [21]. The most recent systematic review on the epidemiology of FC in children has shown that 0.7 to 28.8% are suffering from FC [1]. However that review did not report separate prevalence rates for young children. Recently, van Tilburg et al. reported a prevalence of FC of 4.7 and 9.4% in respectively for infants and toddlers living in the USA [3]. A similar study from Thailand including children of 4 months to 5 years of age found a much lower prevalence (1.6%) [22]. Higher prevalence rates in children aged between 3 and 5 years are reported Hong Kong (28.8%) [23] and The Netherlands (12%) [24].

In this cohort of Sri Lankan infants there was a higher prevalence rate of constipation (8.3%) compared to infants from the USA (4.7%) and those from Thailand (1.6%) [3, 22]. The peak age of developing constipation in Sri Lankan children was 3–4 years while in Thailand this was 2–3 years. The highest prevalence of FC in children aged between 3 and 5 years was reported in Hong Kong (28.8%) [23]. Our prevalence rate of 6.9% around 2–3 years is lower than the data from Thailand (7.2%), USA (9.4%) and The Netherlands (12%). All studies (except the study from Hong Kong which used Rome II criteria) have used the Rome III criteria for the diagnosis of constipation and collected data from parents of young children. The wide variation in prevalence could be attributed to variation in dietary patterns, cultural differences in toilet training, differences in child rearing, and perhaps other social determinants unknown to us. International collaborative studies using the same methods and defined age groups are needed to generate a clearer global picture of the epidemiology of FC in young children.

In this study we found a large percentage of young children with constipation with a history of painful or hard bowel motions (94%) and a history of large diameter stools (66%). This is in accordance with a previous study conducted in Sri Lanka in older children in which 71% of children had a history of painful or hard bowel motions and 66% had large diameter stools [13]. Studying a large cohort of children similar to our sample, Loening-Baucke reported painful defecation as the most frequently reported symptom (67%) [25]. Another study reported painful defecation and hard stools in 43 and 92% infants and young children respectively [26]. Surprisingly, none of the children in this study suffered of fecal incontinence. Generally, faecal incontinence is a feature of severe constipation. Lack of faecal incontinence suggests that most of these children may have suffered from mild constipation.

We studied a number of potential socio-demographic risk factors that could be associated with FC. Living in an urban area of the district was the only significant factor associated with FC in this cohort of Sri Lankan children. This finding is in accordance with our previous findings, where older children living in urban areas of Sri Lanka had a higher tendency to develop FC than their rural counterparts [13]. Ludvigsson has made a similar observation in children living in Sweden [27]. Although previous studies in older children have noted an association between gender and development of constipation [13], our data did not support this. It has been suggested that the differential prevalence of FGDs are related to differences in sex hormones in adults [28]. The fact that these hormonal profiles are not well established in infants and young children would explain the lack of difference between girls and boys.

Associations have been described between constipation and other FGDs in children and obesity and being overweight [29]. A hospital-based study on children with morbid obesity reported a delay in colonic transit confirming constipation in these children [30]. Moreover, in young children attending daycare centers in Korea, constipation was significantly associated with 2 h or less of outdoor play activities per day, and three or fewer servings of vegetables and fruits per day [21].

Contrary to these findings, our data showed that overweight or obesity, was not a risk factor for FC. A recent study on school children in Colombia confirms our findings [31]. However, this may be due to the small number of children with obesity/overweight in our sample. For the first time, we noted that children with underweight have a higher tendency to develop FC. It is perhaps necessary to explore the possibility of abnormalities in transit and anorectal function in underweight children with constipation as well.

In a previous study, we noted home-related stress and abuse to predispose children to develop FC [12]. We found that children subject to violence developed significantly more FC, but this correlation was not confirmed by logistic regression analysis. Studies among adults have also found that facing adverse life events as young children are a risk factor to develop IBS in adulthood [32]. Therefore, we hypothesized stressful life events could predispose young children to develop FC. However, contrary to our hypothesis, home related stresses were not associated with FC in infants and young children. Similar to our findings, studying young children (7–48 months) with constipation living in the West bank, Gaza strip and Jordan, Froon-Torenstra and co-workers noted that stressful life events had not contributed to the development of constipation [33]. Economic crises in the family and change of residence were also not associated with FC. Previous studies in adults and young children suggest that the brain-gut-axis plays an important role in developing FGDs after facing stresses and abuse [34]. Our finding of a lack of significant association between stress and abuse and development of FC in young children may be due to several reasons. First, the brain-gut-axis of young children may not be fully mature to appreciate the stresses, so that the alterations that lead to the development of FGDs are minimal. Second, a time lag may be necessary to develop FGDs after exposure to adverse life events. Over-emphasis of these events by adults with severe FGDs and possible recall bias in those retrospective studies are also possible reasons. In addition, we had to rely on mothers to collect information regarding home related stresses. Whether these factors truly lead to stress that can alter the brain-gut axis, leading to the development of constipation in young children, need further studies, including long-term follow-up of children faced with adverse life events and ill-treatment.

The majority of infants and young children with FC have received treatment for their symptoms. However, only 24% of young children were seen by a medical doctor. These consultation rates are better than a previous study in Sri Lanka on older children, which reported that only 3% of the affected children and adolescents sought medical advice for their symptoms [35]. It is possible that parents of young children are more conscious of abnormal bowel habits of their children than parents of older children who are toilet trained and independent. Adolescents are also reluctant to discuss their bowel habits with their parents. This is a possible reason for the lower healthcare consultation in the previous study.

We were able to categorize the treatment modalities into 3 main groups. They include: dietary advice, non-pharmacological modification and laxatives. Recent guidelines have clearly indicated the lack of therapeutic efficacy of increasing dietary fiber and consumption of water in the management of FC in children [36, 37]. Despite these facts, nearly half of the children received dietary interventions as primary treatment. Poor toilet training is recognized as a potential risk factor for FC especially in young children [10]. However, only 32% of children in this study received advice on toilet training. Oral laxatives are the currently recommended first line treatment for FC by both National Institute of Clinical Excellence (NICE), UK and combined European and North American Societies of Pediatric Gastroenterology, Hepatology and Nutrition Guidelines. Surprisingly, only one third has received oral laxative as therapy. Therefore, it is imperative to recognize that a nationwide educational program is essential to educate medical practitioners as well as the lay public about effective treatment options and possible consequences of poorly treated constipation.

There are several strengths in our study. We used a large sample of infants and young children in this study. Therefore, our findings could be generalized to the entire country. Since our study was based on clinics conducted by trained medical personnel, organic causes could be excluded by reviewing the child health development records. We used widely accepted Rome III criteria to define FC in infants and young children.

However, there are limitations to our study as well. There was no suitable questionnaire to assess symptoms of FGDs in infants and young children at the time we conducted the study. Therefore, we created the questionnaire by using the bowel habits questions from the QPGS questionnaire of children and adolescents. The QPGS questionnaire is a widely used and accepted way of assessing bowel habits of older children and its validated version is available in native language of Sri Lanka (Sinhala). Our group has used it in previous studies successfully [38–40]. We relied on mothers to obtain details of bowel habits of their children and used a self-administered questionnaire, which could have led to exaggeration of symptoms, especially when mothers are suffering from FGDs themselves. In accordance with other studies reporting on prevalence of FGDs in young and older children a rectal examination was not performed, mainly because of ethical reasons [3, 13]. This might underestimate the prevalence of FC in children.

Our study has several implications. Firstly, the data show that FC is an important health problem in younger children as well. The lack of association of stressful life events, which are currently considered as important risk factors, and FC in this age group, compared to the older children possibly indicates that there is potentially a lag period between exposure to stress and development of FC. This window period could be used to manipulate the brain-gut-axis to reduce the risk of developing FGDs in children with early interventions. However, this concept needs further exploration. We also found that at least one third of children with FC are treated with ineffective therapeutic modalities. This demands the institution of a sustainable awareness program to educate medical practitioners as well as the general public.

## Conclusion

FC is a common clinical problem in infants and young children in Sri Lanka. Living in urban areas and being underweight for the age are significantly and independently associated with FC. Contrary to previous findings in older children, stressful life events do not significantly predispose young children to develop FC. One quarter of children received treatment by a medical doctor for their symptoms and a large proportion of infants and young children received ineffective therapeutic interventions according to currently accepted guidelines.

## Additional file

Additional file 1: Questionnaire used for data collection. (PDF 122 kb)

## Data Availability

The datasets used and/or analyzed during the current study are available from the corresponding author on reasonable request.

## Abbreviations

CHDR: Child Health Development Record
FC: Functional Constipation
FGD: Functional Gastrointestinal Disorder
MOH: Medical Officer of Health
NICE: National Institute of Clinical Excellence
QPGS: Questionnaire on Pediatric Functional Gastrointestinal Symptoms
