# Supplementary material for: Functional constipation in infancy and early childhood: epidemiology, risk factors, and healthcare consultation
Source: BMC Pediatr. 2019 Aug 15;19:285. doi: 10.1186/s12887-019-1652-y (PMC6694472; doi:10.1186/s12887-019-1652-y)
Supplement: Supplementary file 1 — Questionnaire used for data collection. (PDF 122 kb) [file 12887_2019_1652_MOESM1_ESM.pdf]

Case number:……………………….

Assessment of constipation among Sri Lankan children aged 7 months to 4 years

Date:……………………….................   Child Health Record No ....
Health Center...
Location: ___ …, ____ ….., ____ ……
Age categories: ___ 7-12 mo, ___ 13-24 mo, ___25-36 mo, ___ 37-48 mo

Part 1 and 2 of the questionnaire will be completed by interview of the mother.
Part 3 and 4 of the questionnaire are self-administered and will be completed by the mother.

PART 1

Information about the child:

1. Name : ....
2. Date of Birth : ....
3. Sex : ___ male, ___ female
4. How many siblings : number of females…………number of males.............
5. What is the birth order? : ....
6. Current weight : ……………………………… grams
7. Current height : ………………………………. cm
8. a. Growthcurve (see Child Health Record)? ____ normal, ___ abnormal
   b. If abnormal, which growthcurve (see Child Health Record)
   1. ___ overweight (> 2SD weight for length)
   2. ___ underweight (< 2SD weight for age)
   3. ___ stunting (< 2 SD length for age)
   4. ___ wasted (< 2SD weight for length)

9. General medical history : ……………………………………………………………………………
10. Is your child mentally developing normal: __ no, __ yes

PART 2

This section asks about your child's bowel movements. There are many words for bowel movements, such as "poop," "stool," "BMs," and "going to the bathroom for number 2." Your family may use another special word when they talk about poops.

11. In the last 2 months, how often did your child usually have poop?
    1. __ 2 times a week or less → continue with question 12
    2. __ 3 to 6 times a week
    3. __ Once a day
    4. __ 2 to 3 times a day
    5. __ More than 3 times a day

    (2–5: continue with question 13)

12. How long has your child had bowel movements 2 times a week or less?
    1. __ Since less than 2 month
    2. __ Since 2 to 6 months
    3. __ Since 6 months to one year
    4. __ Since 1 - 2 years
    5. __ Since 2 - 3 years
    6. __ Since 3 - 4 years

13. In the last 2 months, what was your child's poop usually like?
    1. __ Type 1: Very hard (separate hard lumps)
    2. __ Type 2: Hard
    3. __ Type 3: Dry and lumpy
    4. __ Type 4: Smooth and soft
    5. __ Type 5: Very soft blobs
    6. __ Type 6: Mushy
    7. __ Type 7: Watery
    8. __ It depends (his/her poops are not always the same)

14. A. Since how long does your child passes loose/watery stool >3/day?
    1. __Never
    2. __<2 months
    3. __2-6 months
    4. __6 months -1 year
    5. __1-2 year
    6. __2-3 year

    B. This stool pattern started when the child was …months of age
    1. __<6 months
    2. __6-12 months
    3. __13-24 months
    4. __25-36 months

5. __older than than 36 months

C. At what time of the day this stool pattern was noticed?
1. __When the child is awake
2. __Occures during sleep

15. In the last 2 months, did your child have to strain during bowel movements?
    1. __ Never
    2. __ Once in two months
    3. __ 1 to 3 times per month
    4. __ Once a week
    5. __ More than once a week
    6. __ Every day

16. A. In the last 2 months, did your child have pain during defecation?
    7. __ Never
    8. __ Once in two months
    9. __ 1 to 3 times per month
    10. __ Once a week
    11. __ More than once a week
    12. __ Every day

17. In the last 2 months, did your child have a poop that was so big that it clogged the toilet (large diameter stool)?
    1. __ No
    2. __ Yes

18. Some children hold in their poop even when there is a toilet available. They may do this by stiffening their bodies or crossing their legs.
    In the last 2 months, when at home, how often did your child try to hold in a poop?
    1. __ Never
    2. __ Once in two months
    3. __ 1 to 3 times per month
    4. __ Once a week
    5. __ More than once a week
    6. __ Every day

19. a. Is your child toilet trained (clean) at home and outside home?
    1. __ No → continue with question 21
    2. __ No, only at home → continue with question 21
    3. __ Yes

    b. If yes, at what age did your child became clean? ………………………………months

20. For children who became completely toilet trained (clean).
    In the last 2 months, how often was your child's underwear stained or soiled with poop?
    1. __ Never
    2. __ Once in two months
    3. __ 1 to 3 times per month
    4. __ Once a week
    5. __ More than once a week
    6. __ Every day

21. For children who became completely toilet trained (clean).
    In the last 2 months, how often was your child's underwear soiled with urine?
    1. __ Never
    2. __ Once in two months
    3. __ 1 to 3 times per month
    4. __ Once a week
    5. __ More than once a week
    6. __ Every day

22. In the last 2 months, how often did your child pass blood with stools?
    1. __ Never
    2. __ Once in two months
    3. __ 1 to 3 times per month
    4. __ Once a week
    5. __ More than once a week
    6. __ Every day

Did your child have the following symptoms during the last 2 months?

23. Abdominal pain	1. __ no	2. __ yes
24. Vomiting	1. __ no	2. __ yes
25. Loss of appetite	1. __ no	2. __ yes
26. Loss of weight	1. __ no	2. __ yes

27. In your opinion, is the stool pattern a problem for your child?
    1. ___ No
    2. ___Yes

28. Did a doctor or nurse ever examine your child and say that your child had a huge poop inside?
    1. __ No
    2. __ Yes

29. a. Did you ever visit an UNRWA health center because your child had stool problems?
    1. __ Yes
    2. __ No, we went to another clinic or hospital → *continue with question 33*
    3. __ No → *continue with question 33*

b. If yes, at what age?..................................months.

c. If yes, what tests did the doctor/nurse do (more answers are possible)?
    1. __ Examination abdomen
    2. __ Anal and rectal exam
    3. __ Blood test :……………………………………
    4. __ X-ray :……………………………………
    5. __ ultrasound :……………………………………
    6. __ other :……………………………………
    7. __ no test

30. a. Has your child ever been treated for constipation by an UNRWA doctor/nurse?
    1. __ Yes
    2. __ No
    3. __ No, self treatment or pharmacy advise

b. The treatment included (more answers are possible):
    1. __ dietary advice:
        a. __ eating more fibres
        b. __ eating more fruits
        c. __ drinking more water
        d. ___ other…………………………………………
    2. __ child behavior advice:
        a. __ toilet training,
        b. __ punishing
        c. __ rewarding
        d. __ other…………………………..
    3. __ oral laxatives:
        a. ___ tablets form ……………………………………….
        b. ___ syrup form …………………………………………
        c. ___ powder form ………………………………………
    4. __ rectal laxatives:
        a. ___ glycerin supp
        b. ___ other supp
        c. ___ rectal enema

5. __ herbal remedies, specify……………………………………
6. __ other……………………………………………………………

30. In the last two months, were you subject to physical or verbal violence in your own house?
    1. __ Never
    2. __ Once in two months
    3. __ Once a month
    4. __ More than once a month
    5. __ Once a week
    6. __ More than once a week
    7. __ Daily

31. In the last two months, was your child subject to physical or verbal violence in your own house?
    1. __ Never
    2. __ Once in two months
    3. __ Once a month
    4. __ More than once a month
    5. __ Once a week
    6. __ More than once a week
    7. __ Daily

PART 3

Information about the family

32. Mother's age in years :…………………………………………………………….
33. Mother's marital age in years :…………………………………………………………….
34. Mother's years of education :…………………………………………………………….
35. Mother's employment:
    1. Unemployed
    2. Unskilled worker
    3. Skilled (hand)worker
    4. Civil employee
    5. Merchant
    6. Professional with a degree
    7. Other occupation…………………………………………

36. Father's age in years :…………………………………………………………….
37. Father's marital age in years :…………………………………………………………….
38. Father's years of education :…………………………………………………………….
39. Father's employment:
    1. Unemployed
    2. Unskilled worker
    3. Skilled (hand)worker
    4. Civil employee
    5. Merchant
    6. Professional with a degree
    7. Other occupation…………………………………………

40. Household income:

M.A. Benninga 23-10-14 16:18
Opmerking: Don't know what to fill in here

1. ___ less than
2. ___
3. ___ more than

41. Does your income meet your essential needs?
    1. ___ no
    2. ___ yes

42. Does your family have any loans?
    1. ___ no
    2. ___ yes

43. How is the relationship with your husband and family members who are living in your house?
    1. ___ Very good
    2. ___ Good
    3. ___ Bad
    4. ___ Very bad

44. Place of residence:
    1. ___ urban area
    2. ___ rural area

45. a. Was your family forced to change their place of residence because of security reasons or other related reasons?
    1. ___ no
    2. ___ yes

    b. If yes, in which year ? …………….

Case number:………………………..

PART 4

M.A. Benninga 23-10-14 16:19
Opmerking: These questions were asked to people on the westbank and in Jordan

Information about exposure to traumatic events.

Did your child experience any of the items below in the last year?
Please check 'yes' or 'no' for each item.

46. Has your child seen mutilated bodies and wounded people on television ___ no, ___ yes
47. Has your child witnessed day raids of your family house ___ no, ___ yes
48. Has your child witnessed night raids of your family house ___ no, ___ yes
49. Has your child witnessed firing at your house by tanks and heavy artillery ___ no, ___ yes
50. Has your child witnessed demolition of your family house ___ no, ___ yes
51. Has your child witnessed shooting of a close relative ___ no, ___ yes
52. Has your child witnessed beating of a close relative ___ no, ___ yes
53. Has your child heard the killing of a close relative ___ no, ___ yes
54. Has your child witnessed the killing of a close relative ___ no, ___ yes
55. Has your child witnessed the arrest of a close relative ___ no, ___ yes
56. Has your child witnessed firing at neighbor's house by tanks and heavy artillery ___ no, ___ yes
57. Has your child witnessed shooting of a neighbor ___ no, ___ yes
58. Has your child heard the killing of a neighbor ___ no, ___ yes
59. Has your child witnessed the killing of a neighbor ___ no, ___ yes
60. Has your child witnessed demolition of a friend's house ___ no, ___ yes
61. Has your child witnessed beating of a friend ___ no, ___ yes
62. Has your child witnessed bombardment of other people's houses by airplanes, helicopters or drones ___ no, ___ yes
